# Relationship Between Difficulties in Daily Activities and Falling: Loco-Check as a Self-Assessment of Fall Risk

**DOI:** 10.2196/ijmr.5590

**Published:** 2016-06-20

**Authors:** Manabu Akahane, Akie Maeyashiki, Shingo Yoshihara, Yasuhito Tanaka, Tomoaki Imamura

**Affiliations:** ^1^ Faculty of Medicine Department of Public Health, Health Management and Policy Nara Medical University Kashihara Japan; ^2^ Faculty of Medicine Department of Orthopaedic Surgery Nara Medical University Kashihara Japan

**Keywords:** accidental falls, disability evaluation, self-assessment, activity of daily living

## Abstract

**Background:**

People aged 65 years or older accounted for 25.1% of the Japanese population in 2013, and this characterizes the country as a “super-aging society.” With increased aging, fall-related injuries are becoming important in Japan, because such injuries underlie the necessity for nursing care services. If people could evaluate their risk of falling using a simple self-check test, they would be able to take preventive measures such as exercise, muscle training, walking with a cane, or renovation of their surroundings to remove impediments. Loco-check is a checklist measure of early locomotive syndrome (circumstances in which elderly people need nursing care service or are at high risk of requiring the service within a short time), prepared by the Japanese Orthopaedic Association (JOA) in 2007, but it is unclear if there is any association between this measure and falls.

**Objective:**

To investigate the association between falls during the previous year and the 7 “loco-check” daily activity items and the total number of items endorsed, and sleep duration.

**Methods:**

We conducted an Internet panel survey. Subjects were 624 persons aged between 30 and 90 years. The general health condition of the participants, including their experience of falling, daily activities, and sleep duration, was investigated. A multivariate analysis was carried out using logistic regression to investigate the relationship between falls in the previous year and difficulties with specific daily activities and total number of difficulties (loco-check) endorsed, and sleep duration, adjusting for sex and age.

**Results:**

One-fourth of participants (157 persons) experienced at least one fall during the previous year. Fall rate of females (94/312: 30.1%) was significantly higher than that of males (63/312: 20.2%). Fall rate of persons aged more than 65 years (80/242: 33.1%) was significantly higher than that of younger persons (77/382: 20.2%). Logistic regression analysis revealed that daily activities such as “impossibility of getting across the road at a crossing before the traffic light changes” are significantly related to falling. Logistic regression analysis also demonstrated a relationship between the number of items endorsed on loco-check and incidence of falling, wherein persons who endorsed 4 or more items appear to be at higher risk for falls. However, logistic regression found no significant relationship between sleep duration and falling.

**Conclusions:**

Our study demonstrated a relationship between the number of loco-check items endorsed and the incidence of falling in the previous year. Endorsement of 4 or more items appeared to signal a high risk for falls. The short self-administered checklist can be a valuable tool for assessing the risk of falling and for initiating preventive measures.

## Introduction

People aged 65 years or older accounted for 25.1% of the Japanese population in 2013 [1], and this characterizes the country as a “super-aging society.” As the Japanese society ages, fall-related injuries are becoming a greater concern. Falls cause severe injuries such as femoral neck fractures and compression fractures of vertebrae, leading to disability in daily life [2-4]. Aging is also related to increased risk of musculoskeletal problems such as lumbar stenosis and osteoarthritis in knee and hip joints [5,6]. These musculoskeletal problems, including fall-related injuries, underlie the necessity for nursing care services in approximately 21% of cases receiving such services among elderly people in Japan [5,7].

“Locomotive syndrome,” which was proposed by the Japanese Orthopaedic Association in 2007, refers to circumstances in which elderly people need nursing care service or are at high risk of requiring the service within a short time [7,8]. Locomotive syndrome is, in part, due to diseases of the locomotive organs such as osteoarthritis, spinal canal stenosis, osteoporosis, and rheumatoid arthritis, and is associated with symptoms that include pain, limitations in the range of joint movement, reduced balance capability, and slow pace of walking, as well as frequent falling. A self-check tool called “loco-check” can assess whether a person is at risk of locomotive syndrome. Loco-check comprises 7 items regarding daily activities and is an acceptable measure for detecting early-stage locomotive syndrome [8]. Because the public health burden of fractures by accidental falls is increasing, studies of fall prevention and risk assessment have been a major focus of public health and nursing care [9]. To prevent falls and subsequent disability in daily life, measures such as exercise, muscle training, walking with a cane, or renovating one’s surroundings to remove impediments are important [10-12]. Risk assessment tools are required for screening those at risk of falls. In this study, we focused on the association between the number of loco-check items endorsed and falling.

## Methods

### Survey Method and Subjects

This study was conducted with the approval of the Ethics Committee of Nara Medical University (authorization code: 335). The general health condition of the participants, including their experience of falling and their loco-check data, was investigated with an Internet-based questionnaire in April of 2011. We conducted the survey using an Internet panel survey company. All respondents were registered as panel members with the company.

The panel survey in this study included registrants aged between 30 and 90 years. Participants were stratified into 3 age groups that spanned 20-year categories (30-49, 50-69, and 70-90 years). Each group included 208 participants (104 males and 104 females), for a total sample of 624. The survey was closed when the number of participants in each group achieved the target sample size. Registrants completed and transmitted their responses via the website.

### Questionnaire for Degree of Recognition

Demographic characteristics of participants, such as age, sex, educational background, occupation, and residential area were already recorded during their registration as a member of the firm’s Internet panel. The first question specific to our study asked whether participants were familiar with locomotive syndrome, metabolic syndrome, and cognitive impairment, to determine the recognition rates for these conditions. The survey also inquired about the medical conditions of participants, their falling experiences, and loco-check items, as described in the following sections.

### Self-Assessment Using Loco-Check

The loco-check checklist for locomotive syndrome was prepared by the Japanese Orthopaedic Association (JOA) in 2007 [8]. According to the JOA proposal, a participant who endorses at least one of the 7 statements on the checklist may have locomotive syndrome. The 7 categories are as follows: (1) you cannot put on a pair of socks while standing on one leg; (2) you stumble or slip in your home; (3) you need to use a handrail when going upstairs; (4) you cannot get across the road at a crossing before the traffic light changes; (5) you have difficulty walking continuously for 15 minutes; (6) you find it difficult to walk home carrying a shopping bag weighing about 2 kg (eg, two 1-L milk cartons); and (7) you find it difficult to do housework requiring physical strength (eg, use of a vacuum cleaner, moving futons into and out of a closet).

### Survey of Falling Experience, Sleep Duration, and Medical History

We asked about falling experiences and sleep duration over the previous year. Sleep duration was a multiple-choice item with 4 response options (6 hours or less, 7 hours, 8 hours, and 9 hours or more). Personal medical history items asked whether the participant had osteoarthritis of the knee or hip joints, spondylosis deformans, spinal canal stenosis, osteoporosis, low back pain, slipped disk, rheumatoid arthritis, cerebral infarction, stroke, brain tumor, or myocardial infarction.

### Statistical Analysis

The recognition rate for locomotive syndrome was calculated and compared with those for metabolic syndrome and cognitive impairment. Number of falls was compared between persons with and without disease of locomotive organs using a chi-square test.

A logistic regression analysis was conducted. The presence or absence of falls in the past year was the dependent variable. The independent variables included sex, age category, and presence or absence of endorsement of the 7 loco-check items. We also carried out a separate logistic regression analysis identical to the first with the exception that the total number of loco-check items endorsed was recoded into 3 categories (0, 1-3, and ≥4). The relationship between sleep duration and falling experience was analyzed by logistic regression analysis adjusted for sex and age.

The statistical analyses were conducted with SPSS version 21.0 (IBM, Chicago, IL, USA). The level of significance was set at *P*<.05.

## Results

### Baseline Characteristics and Recognition Rates

The mean and standard deviation (SD) for age of participants was 58.5 (SD 16.2) years and 58.5 (SD 16.5) years in male and female participants, respectively. Height and weight of the male group were significantly higher than those of the female group (mean 168.5, SD 6.4 cm vs mean 155.5, SD 5.9 cm; mean 66.8, SD 12.9 kg vs mean 52.0, SD 7.6 kg; *P*<.001). Prevalence of personal medical history was as follows: osteoarthritis of knee (22/624: 3.5%) or hip (10/624: 1.6%), spondylosis deformans (10/624: 1.6%), spinal canal stenosis (21/624: 3.4%), osteoporosis (29/624: 4.6%), low back pain (75/624: 12.0%), slipped disk (38/624: 6.1%), rheumatoid arthritis (13/624: 2.1%), cerebral infarction (19/624: 3.0%), stroke (4/624: 0.6%), brain tumor (2/624: 0.3%), and myocardial infarction (13/624: 2.1%).

Recognition rates for locomotive syndrome, metabolic syndrome, and cognitive impairment were 6.3% (39/624), 84.9% (530/624), and 87.3% (545/624), respectively.

### Falling Experience in the Previous Year

**Figure 1:**
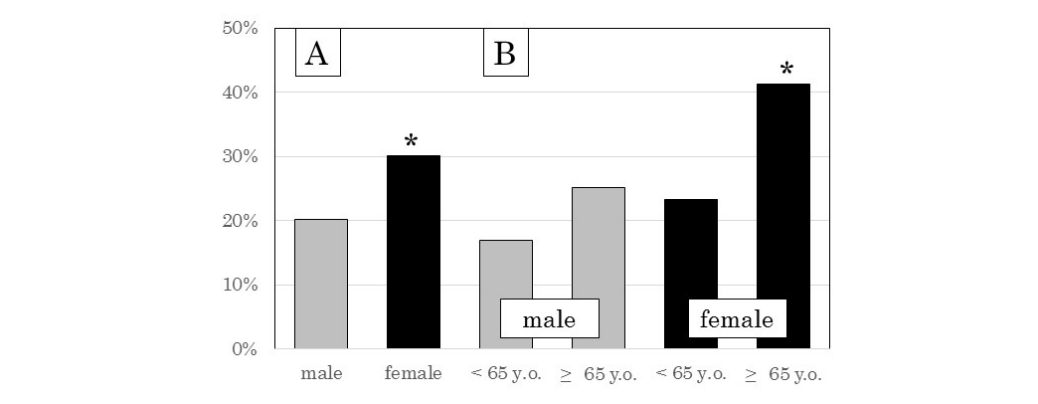
A. Percentage of males and females who experienced falls. B. Percentage of participants who had fallen, by age and sex. *Statistical significance (*P*< .05).

A total of 157 participants (157/624: 25.2%) had experienced at least one fall in the previous year. The percentage of females (94/312: 30.1%) who had fallen was significantly higher than that of males (63/312: 20.2%, *P*=.004, Figure 1, part A). The percentage of those 65 years and older who had fallen (80/242: 33.1%) was significantly higher than that of younger persons (77/382: 20.2%, *P*<.001); however, this result appeared to be attributable mostly to differences among older and younger females (49/119: 41.2% vs 45/193: 23.3%, *P*=.001 for females; 31/123: 25.2% vs 32/189: 16.9%, *P*=.08 for males; Figure 1, part B).

### Percentage of Endorsements for Each Item of the Checklist

**Figure 2:**
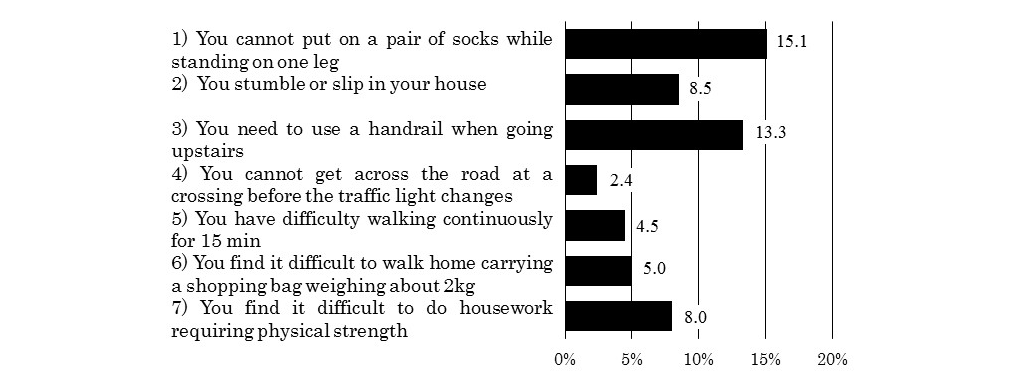
Percentage of participants who endorsed each item of the self-assessment checklist.

Percentages of endorsement by item are shown in Figure 2. The percentage of participants who endorsed at least one item of the checklist was 23.2% (145/624).

### Falling Experience According to Disease of Locomotive Organs

**Figure 3:**
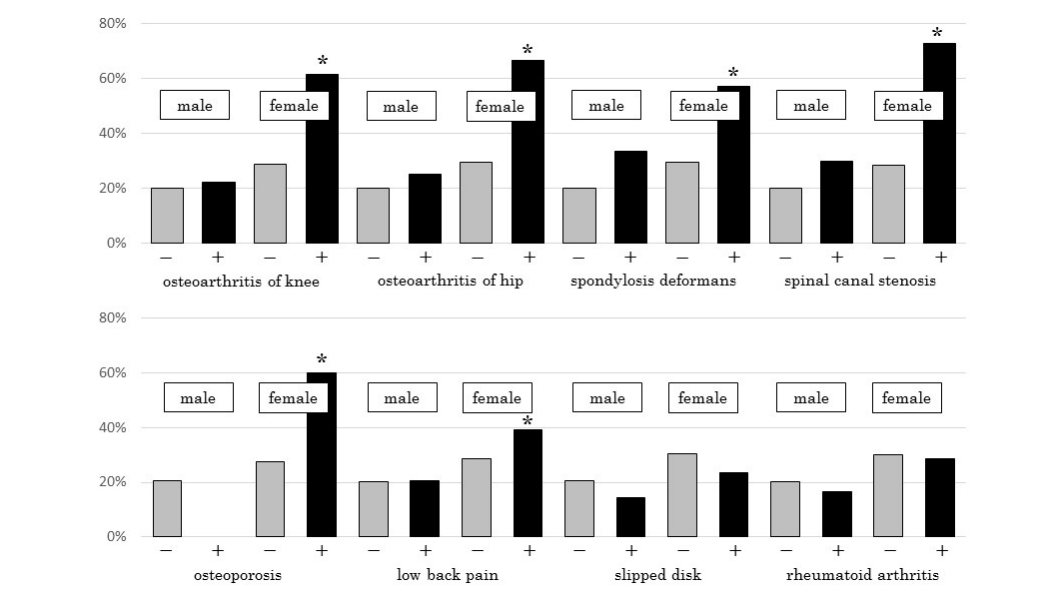
Incidence of falling among participants with diseases of locomotive organs. *Statistical significance (*P*<.05).

The incidence of falling among persons who had disease of locomotive organs is shown in Figure 3. There was no significant difference among males with and without disease of locomotive organs. In contrast, females who had osteoarthritis of the knee or hip, spondylosis deformans, spinal canal stenosis, osteoporosis, or low back pain had a significantly higher incidence of falling in the previous year compared with females who did not have these diseases of locomotive organs.

### Self-Assessment Checklist and Falling Experience

Logistic regression analysis revealed a significant relationship between all but one of the 7 loco-check items and falling. As shown in Table 1, particularly high odds ratios were found for items 2, “You stumble or slip in your home,” and 4, “You cannot get across the road at a crossing before the traffic light changes.” No significant relationship was found for item 6, “You find it difficult to walk home carrying a shopping bag weighing about 2 kg (eg, two 1-L milk cartons).”

### Number of Items Endorsed, Sleep Duration, and Falling

Logistic regression analysis revealed a significant relationship between 2 of the 3 categorical measures of loco-check items endorsed (0, 1 to 3, or ≥4 items) and falling. As shown in Table 2, endorsing 1-3 items or ≥4 items predicted falling, but endorsing 0 items did not. Logistic regression analysis for sleep duration demonstrated that there was no significant relation between this variable and falling (Table 3).

**Table 1:** Logistic regression analysis for the 7 loco-check items.

Checklist items	OR^a^	95% CI	*P* value^b^
1. You cannot put on a pair of socks while standing on one leg	3.25	1.98-5.33	<.001
2. You stumble or slip in your house	8.81	4.53-17.18	<.001
3. You need to use a handrail when going upstairs	2.95	1.74-4.99	<.001
4. You cannot get across the road at a crossing before the traffic light changes	8.13	2.17-30.47	<.001
5. You have difficulty walking continuously for 15 minutes	2.42	1.07-5.50	.03
6. You find it difficult to walk home carrying a shopping bag weighing about 2 kg	2.02	0.93-4.37	.08
7. You find it difficult to do housework requiring physical strength	3.53	1.86-6.71	<.001

^a^ OR: odds ratio.

^b^ Statistical significance (*P*<.05).

**Table 2:** Logistic regression analysis for accumulation of 7 loco-check items.

Independent variables		Odds ratio	95% CI	*P* value^a^
**Sex**		1.60	1.08-2.36	.19
**Age**		1.01	0.99-1.02	.32
**Accumulation of items**				
	0	Reference		
	1-3	3.21	2.04-5.12	<.001
	≥4	10.36	4.00-26.85	<.001

^a^ Statistical significance (*P*<.05).

**Table 3:** Logistic regression analysis for sleep duration.

Independent variables		Odds ratio	95% CI	*P* value^a^
**Sex**		1.78	1.22-2.59	<.001
**Age**		1.03	1.01-1.04	<.001
**Sleep duration, hours**				
	≤6	Reference		
	7	1.10	0.71-1.71	.68
	8	1.22	0.75-2.00	.43
	≥9	0.57	0.18-1.81	.34

^a^ Statistical significance (*P*<.05).

## Discussion

### Overall

Our study demonstrated a reliable relationship between the number of loco-check items endorsed and incidence of falling, indicating that persons who endorsed 4 or more items appear to be at high risk for falls. Those who have difficulty in certain daily activities such as being able to get across the road before the traffic light changes may be at particularly high risk. A previous study reported that older people recognize falls as a serious threat and that information dissemination, along with fall risk self-assessment, may be a low-cost way to improve identification of fall risk and stimulate fall-prevention activities [13]. Although evaluating fall risk using self-check items is becoming common [13,14], it is still problematic because the checklists tend to be lengthy. In contrast, loco-check is easy to use because it has only 7 items, all of which describe familiar daily activities. To our knowledge, ours are the first data to show that loco-check is useful as a self-assessment tool for evaluating fall risk.

### Critical Health Problem by Falling

Falling is an event that often results in critical health problems such as femoral neck fracture or compression fractures of vertebrae; therefore, falls remain a major public health problem among people aged 65 years or older. Some elderly people require nursing care services as a result of these problems. There are two categories of risk factors in falls: intrinsic and extrinsic. The former includes an individual’s physical and cognitive abilities such as muscle strength, balance capacity, reactive power, dual tasking, and sleep disturbances; and the latter includes home hazards, improper use of assistive devices, and inappropriate footwear [9]. Behavior-related risk factors are hurrying, risk-taking, and physical inactivity [15]. Studies of fall prevention and risk assessment have been a major focus of public health and nursing care [9]. Previous studies have reported that falls at home are more frequent than in nursing care homes [16] and inpatient settings [17]. To prevent falls, it would be ideal for persons at home to evaluate their potential risk by means of a simple self-checklist. Self-assessment of risk factors can help identify individuals who might benefit from interventions aimed at fall prevention. Our results demonstrated that one’s risk of falling can be self-evaluated by a short checklist that consists solely of items about daily activities. Individuals who endorse 4 items or more on the checklist are particularly advised to meet with a physician or physical trainer to treat their musculoskeletal disorders as well as reducing their extrinsic risk factors. One can reduce extrinsic risk factors by removal of environmental hazards, renovation of the home, proper selection of assistive devices, and maintenance of muscular strength and balance through muscle training with activities such as walking, squatting, and balancing.

### Relationship between Sleep Duration and Falling

Insomnia and disturbed sleep are increasingly common for older people [18]. Sleep disturbances cause slowed responses and subsequently result in greater risk of accidents and injuries such as falls and fractures. Falls are a major syndrome in the elderly, in which sleep disturbance may play an important role [18-21]. Although a few studies have reported the relationship between falls and sleep disturbances, we did not find any link between sleep duration and falling. This may be because the relationship between sleep disturbances and risk of falls is mediated by mechanisms such as balance ability, cognitive function, and medication. We used an Internet panel survey in this study; therefore, our participants may have been healthier, with fewer cognitive problems and less depression than is observed in the elderly population in general. Elderly persons sometimes exhibit different circadian rhythms compared with younger individuals, for example, earlier bedtimes and wake-up times. Participants in our study were generally sleeping more than 6 hours per night, and there was not a single participant whose sleep duration was less than 5 hours, indicating that our sample generally slept well.

### Fall Prevention

Fall prevention is a major public health theme because falls occur frequently and can cause subsequently devastating problems for elderly individuals, affecting their morbidity, mortality, and locomotive ability. Falls occur in 30%-60% of older persons each year, and 10%-20% of these result in injury, hospitalization, and/or death [22]. The cause of falls is generally multifactorial, including certain medications, environmental hazards in daily life, and physiological changes due to aging. The most important risk factors are muscle weakness and problems with gait and balance [22]. Environmental barriers are responsible for 30%-50% of falls. At home, thresholds, stairs, carpets, and slippery surfaces represent barriers [23]. Our study indicated that one can assess the risk factors such as muscle weakness and gait and balance problems using the self-administered loco-check for locomotive syndrome. Even elderly persons can easily detect their risk of fall using this tool. Loco-check is simple and easy to understand because its 7 daily activities represent gait and balance abilities. Adding the number of items endorsed is also easy, resulting in a quick assessment of one’s fall risk. Families of high-risk adults could be alerted to remove dangerous barriers at home such as slippery surfaces and carpets, and public health staff could apply interventions such as balance exercises for the high-risk person.

Previous studies have reported an association between locomotive syndrome and health-related quality of life (HRQoL). Iizuka et al [24] reported that endorsing a larger number of items in loco-check was associated with reduced HRQoL as assessed by the EuroQoL-5 dimensions (EQ-5D) and EuroQoL visual analogue scale (EQ VAS). EQ-5D consists of 5 items regarding HRQoL: mobility, self-care, usual activity, pain/discomfort, and anxiety/depression. EQ VAS is a self-reported questionnaire regarding one’s health status. However, neither instrument focuses on the incidence of falls. Seichi et al [25] reported a positive relationship between locomotive syndrome and incidence of falls in the previous year. They used the 25-question Geriatric Locomotive Function Scale (GLFS-25) to identify locomotive syndrome. Others have also reported an association between locomotive syndrome and reduced HRQoL [26]. Our suggested method for assessing fall risk is simply determining the number of items endorsed on the 7-item loco-check self-assessment. Such an assessment would be easy to perform, both for the individual and for public health staff.

### Recognition Rate of Locomotive Syndrome

Participants’ recognition rate of locomotive syndrome was quite low compared with metabolic syndrome and cognitive impairment at the time our survey was conducted in April 2011. The recognition rate has increased dramatically in recent years, for example, it was reported at 36.1% in 2014 [27] and at 44% in 2015 [28]. However, these rates still fall far short of those for metabolic syndrome and cognitive impairment. The Ministry of Health, Labour and Welfare of Japan has set a target recognition rate of 80% by 2022. Major musculoskeletal disorders that may cause locomotive syndrome include osteoarthritis, spondylosis, and osteoporosis. Osteoporosis represents a major public health problem through its association with fragility fractures. The public health burden of osteoporotic fractures is increasing, due in part to an increase in life expectancy. The results of our study indicate that approximately 1 in 4 of our participants (between the ages of 30 and 90 years) could be at risk of locomotive disability, which means that their circumstances could cause them to require nursing care services currently or to be at high risk of requiring such services within a short time.

### Questionnaire Survey and Its Advantages

When we recruited the participants in this study, we created 3 age categories that spanned 20-year categories because the distribution of participant numbers needed to be equivalent among ages. As the actual ages of the participants were recorded, we were able to divide the subjects into 2 groups (<65 years and ≥65 years, as shown in Figure 1) and analyze the relationship between falling experience and daily activities by logistic regression analysis using the actual ages of the participants (as shown in Tables 2 and 3). In general, the risk of falling increases with advancing age [29], and many studies have investigated the fall risk in elderly people only [13,14,26,30]. In contrast, we investigated the fall risk across people of all ages in our study and also analyzed the fall risk of the elderly generation compared with that of the younger generation. It is easy to recognize whether a person is at risk of falling by comparing the fall risk of the elderly generation with that of the younger generation.

### Limitations

This study has a few limitations. First, all measures were based on self-reporting by Internet survey. This may result in some misclassification. Second, we used an Internet panel survey company to collect data from registrants aged between 30 and 90 years. Elderly persons who use computers and the Internet may be more active and healthier than those who do not; therefore, we may have underestimated the incidence of falling. However, Internet panel surveys are becoming common for epidemiology research in the social sciences [31-33]. Third, our study did not find a relationship between sleep duration and falling. However, it is also important to pay attention to sleep quality among older adults, considering the documented impact of sleep disturbances on health [34]. Therefore, in future studies we need to analyze the relationship between falls and sleep quality. Fourth, our study was a survey conducted using an Internet-based questionnaire, therefore we could not justify whether simple self-assessment using loco-check is useful for screening and preventing falls. Concerning these points, further investigation such as prospective cohort study will be needed.

### Conclusions

We conducted an Internet panel survey to investigate the relationship between falls in the previous year and difficulties with specific daily activities, total number of difficulties (loco-check) endorsed, and sleep duration. A multivariate analysis was carried out using logistic regression to investigate the relationship, with adjustments for sex and age. Our study demonstrated a relationship between the number of loco-check items endorsed and the incidence of falling in the previous year. Endorsement of 4 or more items appeared to signal a high risk for falls. To prevent falls, it would be ideal for people at home to evaluate their potential risk by means of a simple self-checklist. The short self-administered checklist of loco-check can be a valuable tool for assessing the risk of falling and for initiating preventive measures.

## Abbreviations

EQ-5D: EuroQoL-5 dimensions
EQ VAS: EuroQoL visual analogue scale
HRQoL: health-related quality of life
JOA: Japanese Orthopaedic Association
SD: standard deviation
